# 

**Published:** 1860-06

**Authors:** 


### CONTENTS.

### JUNE, 1860.

### Lectures, Monographs, and Cases.

PAGE

On Pyæmia. By H. P. Dewees, M.D. Read before the N. Y. Medico-Chirurgical College ........ 817

The Physiology of the Circulation. A Course of Lectures delivered at the College of Physicians and Surgeons, New York, in the Fall Term of 1859. By John C. Dalton, Jr., M.D., Prof. of Physiology and Microscopic Anatomy. Lecture VI ........ 830

Lectures on Displacements of the Uterus. By E. R. Peaslee, M.D., LL.D., Prof. of Obstetrics and Diseases of Women and Children in the New York Medical College. Lecture IV ........ 852

On Coryza Maligna. By Bernard Kelly, M.D., Physician to the New York Dispensary ........ 862

Report of the Committee of the Medical Society of the State of New York on Pharmaceutical Preparations ........ 865

### Monthly Summary of Medical Journalism,

By O. C. Gibbs, M.D., Frewsburg, N. Y.

Reduction of Strangulated Hernia, 872; Ovariotomy, 872; Bloodletting in Inflammation, 872; Yellow Fever, New Treatment for, 873; New Treatment for Congestive Chills, 874; Cause of Goitre, 874; A New Remedy for Consumption, 875; Inversion of the Uterus, 875; Malarial Fever, 876; Trismus Nascentium Treated with Quinine, 878; Treatment of Croup, 878; Subcutaneous Injection, 878; Vomiting in Pregnancy, 879; Hypophosphites in Spurious Hydrocephalus, 879; Sore Nipples, 879; Strangulated Hernia Treated with Opium, 880; Stomatitis Materna, 880; Arsenic in Menorrhagia, Dysmenorrhagia, &c., 881; Fungoid Growth of the Gums and Jaw, 881; Iodide of Potassium in Neuralgia, 881; Iodide of Potassium in Diseases of the Brain in Children, 882.

### Reviews and Bibliography.

Therapeutics and Materia Medica. A Systematic Treatise on the Action and Uses of Medicinal Agents, including their Description and History. By Alfred Stillé, M.D., &c. 2 Vols. Philadelphia: Blanchard & Lea.. 883

A Guide to the Practical Study of Diseases of the Eye, with an Outline of their Medical and Operative Treatment. By James Dixon, F.R.C.S.... 886

### Editorial and Miscellaneous.

To our Readers—Sydenham Society Publications—Garrett on Medical Electricity—Maison de Santé in New York ........ 887

